# Diagnostic Test Accuracy of Serum Anti-PLA2R Autoantibodies and Glomerular PLA2R Antigen for Diagnosing Idiopathic Membranous Nephropathy: An Updated Meta-Analysis

**DOI:** 10.3389/fmed.2018.00101

**Published:** 2018-04-26

**Authors:** Weiying Li, Yuliang Zhao, Ping Fu

**Affiliations:** Division of Nephrology, West China Hospital, Sichuan University, Chengdu, Sichuan, China

**Keywords:** membranous nephropathy, M-type phospholipase A2 receptor, anti-phospholipase A2 receptor, diagnostic test accuracy, secondary membranous nephropathy, sPLA2R, gPLA2R

## Abstract

**Background:**

M-type phospholipase A2 receptor (PLA2R) is known as a major antigen on podocytes, which is involved with the pathogenesis of idiopathic membranous nephropathy (iMN). Many studies have shown that serum anti-PLA2R autoantibodies (sPLA2R) are prevalent in patients with iMN but are rarely detected in secondary membranous nephropathy (SMN) or other glomerulonephritis. The anti-PLA2R is considered as a promising serum biomarker in iMN but reports about its diagnostic value are variable and inconsistent.

**Objective:**

To evaluate the diagnostic test accuracy (DTA) of anti-PLA2R and glomerular PLA2R antigen (gPLA2R) for diagnosing iMN.

**Method:**

MEDLINE, EMBASE, WEB OF SCIENCE, and COCHRANE LIBRARY were searched from 2009 January to February 2018. Heterogeneity was evaluated by *Q* test and *I*^2^. Source of heterogeneity was explored by subgroup analysis and meta-regression. Meta-analysis was executed and reported according to the Preferred Reporting Items for Systematic Reviews and Meta-analyses statement.

**Results:**

Totally, 35 studies were retrieved under the pre-set study eligibility criteria. Twenty-eight studies were included to evaluate the DTA of anti-PLA2R for differentiating iMN from non-iMN. They indicated a pooled sensitivity of 65% (63–67%), specificity of 97% (97–98%), positive likelihood ratio of 15.65 (9.95–24.62), and negative likelihood ratio of 0.37 (0.32–0.42) with a diagnostic OR (sDOR) of 50.41 (31.56 to 80.52) and AUC of 0.9393. No threshold effect was detected. The heterogeneity analysis for sDOR showed that *I*^2^ = 50.3% and Cochran-*Q* = 54.29, df = 27 (*p* = 0.0014). Heterogeneity was significant. Meta-regression revealed that sample size might be the potential source of heterogeneity. Subgroup analysis demonstrated that method type and ratio of patients with nephrotic-range proteinuria at baseline might be the source of heterogeneity. Sixteen studies reported the diagnostic value of glomerular PLA2R antigen for differentiating iMN from non-iMN. The pooled sensitivity, specificity, positive likelihood ratio, negative likelihood ratio, sDOR, and AUC were 79% (76–81%), 90% (88–92%), 8.17 (5.60–11.93), 0.25 (0.19–0.33), 39.37 (22.18–60.13), and 0.9278. Heterogeneity analysis showed that Cochran-*Q* = 35.36; df = 15 (*p* = 0.002), and *I*^2^ for sDOR was 57.6%.

**Conclusion:**

sPLA2R and gPLA2R demonstrated a good diagnostic accuracy in differentiating iMN and non-iMN.

## Introduction

Membranous nephropathy (MN) is a common cause of massive proteinuria, accounting for 20–30% of nephrotic syndrome in Caucasian adults (1). The pathologic findings are featured by diffuse thickening of glomerular basement membrane in the absence of significant hypercellularity and subepithelial deposits of immunoglobulin G (IgG) and complement 3. MN is often idiopathic, and the diagnosis of secondary membranous nephropathy (SMN) is mainly established by the evidence of secondary causes such as systemic lupus erythematosus, hepatitis B infection, malignancies, or the use of certain drugs and characteristic changes in histology. However, the differential diagnosis of idiopathic membranous nephropathy (iMN) and SMN, which is of great clinical significance, could still be challenging in some clinical scenario.

M-type phospholipase A2 receptor (PLA2R) was identified as a major target antigen on glomerular podocytes in iMN. Beck et al. found that serum anti-PLA2R autoantibodies (sPLA2R) were detected in 70% of patients with iMN, and the IgG eluted from renal biopsy tissues of patients with iMN was reactive with PLA2R antigen (2). Emerging evidence from studies about recurrent MN in post-transplantation patients suggested a higher recurrence rate of MN in patients with positive pre-transplantation anti-PLA2R, which gave us more insight into the role of anti-PLA2R in the occurrence of MN (3). Discovery of anti-PLA2R has contributed to substantial advances in our understanding of the pathogenesis of MN. However, the pathogenic effect of anti-PLA2R in iMN has not been verified experimentally yet, and the exact pathogenic role remains to be elucidated. Nevertheless, the significance of sPLA2R and glomerular PLA2R antigen (gPLA2R) in diagnosing and monitoring the disease activity of iMN should not be ignored (4). In this study, we aimed to investigate the diagnostic performance and clinical value of anti-PLA2R autoantibodies as serum biomarkers for diagnosing iMN, which is helpful in assisting the differentiation between iMN and SMN and execution of etiology-based treatment.

## Methods

### Eligibility Criteria

Study eligibility criteria included the following: (1) both cohorts with iMN and non-iMN were enrolled in studies. (2) Index test results for sPLA2R or gPLA2R were reported in cases of iMN and non-iMN. (3) Renal biopsy was used as reference test for diagnosing MN. Identified secondary etiology of MN confirmed that the diagnosis of SMN and exclusion of secondary causes in patients with MN was considered as iMN. (4) Absolute number of true positive, false positive, true negative, and false negative was reported or could be derived. Different methods were used to test the positivity of sPLA2R in studies including ELISA, Western blot (WB), indirect immunofluorescence test (IFFT), and time-resolved fluoroimmunoassay (FIA). All of the studies meeting the eligibility criteria above were included in our meta-analysis, even though different methods for index test might be used.

### Information Sources and Search Strategies

Literature was searched in MEDLINE, EMBASE, WEB OF SCIENCE, and COCHRANE LIBRARY from January 2009 to February 2018. Search strategy in MEDLINE: {[Glomerulonephritis, Membranous (Mesh) OR Membranous Glomerulonephritides (tiab) OR Membranous Glomerulonephritis (tiab) OR Membranous Glomerulopathy (tiab) OR Membranous Nephropathy (tiab) OR Heymann Nephritis (tiab)] AND [Receptors, Phospholipase A2 (Mesh) OR PLA(2) Receptor OR Phospholipase A2 Receptor OR Anti-Phospholipase A2 Receptor OR M-type phospholipase A2 receptor OR anti-PLA2R OR aPLA2R OR PLA2R] AND [Sensitivity and Specificity (Mesh) OR Sensitiv* OR Specific*]}. Search strategy in EMBASE: (“membranous glomerulonephritides” OR “membranous glomerulonephritis”/exp OR “membranous glomerulonephritis” OR “membranous glomerulopathy” OR “membranous nephropathy” OR “heymann nephritis”) AND (“pla2 receptor” OR “phospholipase a2 receptor”/exp OR “phospholipase a2 receptor” OR “anti-phospholipase a2 receptor” OR “m-type phospholipase a2 receptor” OR “anti-pla2r” OR “apla2r” OR “pla2r”) AND (“sensitivity and specificity”/exp OR “sensitivity and specificity” OR “diagnostic value”/exp OR “diagnostic value” OR sensitiv* OR specific* OR “diagnos*”). Search strategy in Cochrane library: {Membranous Glomerulonephritis OR Membranous Glomerulopathy OR Membranous Nephropathy OR [MeSH descriptor: (Glomerulonephritis, Membranous) explode all trees]} AND {PLA (2) Receptor OR Phospholipase A2 Receptor OR M-type phospholipase A2 receptor OR anti-PLA2R OR PLA2R OR [MeSH descriptor: (Receptors, Phospholipase A2) explode all tree]}. The reference lists of the identified articles were also reviewed manually to identify additional articles.

### Study Selection

Two reviewers (MD and MD) screened studies and determined eligibility independently. Disagreements were resolved by discussion and common consensus. Reviewers initially screened the titles and abstracts to detect the potential relevant papers, and then the shortlisted studies were screened again to evaluate their adherence to the eligibility criteria. There was no language restriction for the search but during selection language was restricted to English. Conference abstracts were scrutinized and excluded because they lacked data for quality assessment.

### Data Extraction

Data from all studies were extracted by two reviewers independently (MD and MD) and combined to develop a definitive data collection sheet. Discrepancies during data extraction and methodological quality assessment process were resolved by discussion and global consensus. The extracted information included author, year, country, mean age, gender, study design, sample size, gold standard, method of sPLA2R measurement, time interval between biopsy and antibody measurement, cutoff value, mean serum creatinine, mean albumin, mean 24 h proteinuria, percentage of included patients receiving immunosuppressive therapy (IST) at baseline, percentage of included patients with nephrotic range proteinuria (NRP). Absolute number of true-positive, true-negative, false-positive, and false-negative were retrieved or calculated to develop 2 × 2 contingency table.

### Risk of Bias and Applicability

All the included studies were assessed for their methodological quality and potential sources of bias using Quality Assessment of Diagnostic Accuracy Studies-2 criteria provided by Review Manager (RevMan) Version 5.3.

### Statistical Synthesis and Data Analysis

Sensitivity, specificity, likelihood ratios, diagnostic odds ratios, and receiver operating characteristic curves (sROC) with 95% confidence intervals were pooled using the DerSimonian and Laird method (random-effects model). Studies were categorized according to method type used as an index test and subgroup analysis was conducted to assess the effect of method type on pooled diagnostic performance and study heterogeneity. The level of proteinuria and interference of IST are correlated with the immunologic activity and clinical status, and therefore more subgroup analysis was conducted according to the ratio of patients with NRP at baseline and ratio of patients with IST at baseline. Spearman correlation coefficient of sensitivity and 1 − specificity were calculated to analyze the threshold effect. Publication bias was explored by funnel plot. Asymmetry of the funnel plot was considered to be significant in publication bias. Study heterogeneity was assessed by *Q* test and *I*^2^. *I*^2^ > 50% and *p* value < 0.05 in the *Q* test were interpreted as the presence of significant heterogeneity. Meta-regression and subgroup analysis were conducted to search for the source of heterogeneity. Meta-analysis was executed and reported according to the Preferred Reporting Items for Systematic Reviews and Meta-analyses statement. All the analysis was performed by Meta-DiSc 1.4 and RevMan Version 5.3. Statistical significance was defined as *p* value < 0.05.

## Results

### Search Results

**Figure 1:**
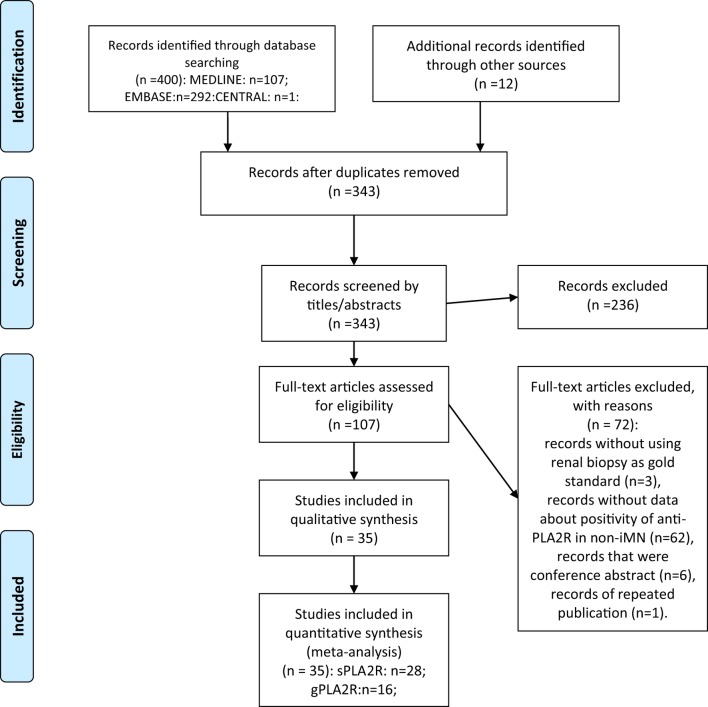
Preferred Reporting Items for Systematic Reviews and Meta-analyses flow diagram.

Overall, 400 records were found from the search strategy, 12 additional records were identified from reference lists of the included records. 343 records were identified after duplicate removal and were screened by titles and abstracts. By reviewing titles and abstracts, 107 records were retrieved for full-text assessment. The following records were excluded: records without using renal biopsy as gold standard (*n* = 3), records with no data about the positive ratio of anti-PLA2R in non-iMN (*n* = 62), records that were conference abstracts (*n* = 6), records of repeated publication (*n* = 1). Thirty-five records were retrieved for quantitative synthesis. Twenty-eight of them reported data about the diagnostic performance of serum anti-PLA2R autoantibodies in differentiating iMN and non-iMN. Sixteen studies provided data about evaluating the value of glomerular PLA2R antigen in diagnosing iMN. The results of the search strategy are presented in the flowchart (Figure 1).

### Study Characteristics

Thirty-five records and 6,085 subjects were enrolled in our analysis. All the patients enrolled in studies had renal biopsy for the pathological diagnosis for MN. SMN was discriminated from iMN by searches for the evidence of secondary causes by clinical evaluation and lab tests. Included studies covered a wide spectrum of country and ethnicity including Caucasian, Asian, Indian and Spanish. Only 20.0% of included studies declared a prospective study design. 51.4% of studies reported that all included patients were sampled at the time of biopsy. 40.0% of studies only included patients whose serum samples were collected before IST. 20.0% of studies only included patients who were presented with nephrotic-range proteinuria at baseline. The baseline characteristics of included studies are presented in Table 1.

**Table 1:** Baseline characteristics of cohorts in all included studies.

Reference	Country	Study design	Sample size	Gender M:F	Age	Time duration	IST ratio	NRP ratio	Median Alb (g/l)	Median 24 h Pr (g/d)	Median sCr (mg/dl)
Beck et al. (2)	USA	NR	97	NR	NR	NR	NR	NR	NR	NR	NR
Qin et al. (5)	China	NR	106	NR	NR	NR	NR	100%	NR	>3.5	NR
Hoxha et al. (6)	German	Retrospective	360	NR	NR	NR	64%	35%	NR	NR	NR
Oh et al. (7)	Korea	NR	123	51/49	54.7 ± 13.9	Concurrent	None	75%	2.7 ± 0.7	Korea0	Korea1
Svobodova et al. (8)	Czech	Retrospective	84	57/27	54.9	30.8% Concurrent	30.8%	43.1%	NR	Czech0	Czech1
Dou et al. (9)	China	Prospective	229	113/116	45.3 ± 15.8	NR	None	NR	2.4 (2.0–2.8)	China0	China1
Akiyama et al. (10)	Japan	Retrospective	131	63/37	67 ± 9	NR	None	59%	2.4 ± 0.7	Japan0	Japan1
Wei et al. (11)	China	NR	148	72/41	48.22 ± 12.74	Concurrent	NR	NR	2.52 ± 0.67	China0	China1
Hihara et al. (12)	Japan	Retrospective	59	27/32	61 (44–68)	Concurrent	None	37.3%	2.6 (2.2–3.4)	Japan0	Japan1
Gopalakrishnan et al. (13)	India	Prospective	75	38/22	44.1 (16–67)	NR	NR	NR	3.04 (1.6–4.6)	India0	India1
Kim et al. (14)	Korea	Prospective	160	53/40	58.20 ± 1.86	NR	69.9%	100%	2.60 ± 0.09	Korea0	Korea1
Hoxha et al. (15)	German	Prospective	88	64/24	56.5 ± 15.8	1.6 ± 2.8 days	None	NR	NR	German0	German1
Hayashi et al. (16)	Japan	Retrospective	25	16/6	64.5 (61–70)	Concurrent	None	NR	2.35 (2.05–3.05)	Japan0	Japan1
Segarra-Medrano et al. (17)	Spain	NR	64	32/15	52.4 ± 15.1	Concurrent	NR	100%	2.3 ± 0.62	Spain0	Spain1
Murtas et al. (18)	Italy	Retrospective	278	121/65	59 ± 16	Concurrent	None	NR	NR	Italy0	Italy1
Ardalan et al. (19)	Iran	Retrospective	30	12/11	34 ± 9.8	NR	96.7%	NR	NR	Iran0	Iran1
Pang et al. (20)	China	Retrospective	705	NR	NR	Concurrent	25.0%	NR	2.7 (2.3–3.1)	China0	China1
Behnert et al. (21)	German	Retrospective	299	NR	NR	Concurrent	None	100%	NR	German0	German1
Hill et al. (22)	Australia	Prospective	40	NR	NR	Concurrent	None	100%	2.19 ± 0.58	Australia0	Australia1
Huang et al. (23)	China	Retrospective	146	NR	NR	NR	NR	NR	NR	China0	China1
Kimura et al. (24)	Japan	Retrospective	44	15/10	61.0 ± 14.4	NR	66%	NR	2.68 ± 0.78	Japan0	Japan1
Li et al. (25)	China	Retrospective	164	42/40	47.44 ± 16.15	<1 wks	NR	50.0%	2.72 ± 2.6	China0	China1
Liu et al. (26)	China	Prospective	141	76/65	44.67 ± 16.06	Concurrent	None	NR	2.41 ± 0.61	China0	China1
Ong et al. (27)	Australia	Prospective	36	NR	59 ± 13.7	<6 months	36%	NR	NR	Australia0	Australia1
Radice et al. (28)	Italy	Retrospective	479	173/79	61(48–70)	NR	None	100%	2.66 ± 0.8	Italy0	Italy1
Timmermans et al. (29)	German	Retrospective	142	69/40	53.7 ± 15.7	Concurrent	NR	NR	NR	German0	German1
Xie et al. (30)	China	Retrospective	267	63/39	NR	Concurrent	None	NR	NR	China0	China1
Zhang et al. (31)	China	Retrospective	458	39/30	55.38 ± 12.6	Concurrent	None	65%	2.15 ± 0. 63	China0	China1
Lonnbro-Widgren et al. (32)	Swiss	Retrospective	79	45/24	52 ± 16	NR	NR	NR	24 ± 8	Swiss0	Swiss1
Larsen et al. (33)	USA	Retrospective	165	56/29	57.5 ± 15.2	NR	NR	NR	NR	USA0	USA1
Yeo et al. (34)	Korea	Retrospective	115	44/15	55.0 ± 13.8	NR	NR	NR	2.7 ± 0.7	Korea0	Korea1
Dong et al. (35)	China	Prospective	248	102/77	49.0 ± 14.3	NR	17.3%	64.1%	2.59 ± 0.72	China0	China1
Gudipati et al. (36)	India	Retrospective	95	47/10	39.1 ± 1.54	NR	NR	NR	NR	India0	India1
Liu et al. (37)	China	Retrospective	252	73/49	52.7 ± 16.9	Concurrent	None	66%	2.58 ± 0.6	China0	China1
Roy et al. (38)	India	Retrospective	153	76/77	41 ± 13.5	NR	NR	100%	2.2 (1.2–4.9)	India0	India1

*IST, immunosuppressive therapy; NRP, nephrotic range proteinuria; NR, not reported; Alb, albumin; 24 h Pr, 24 h proteinuria; sCr, serum creatinine; wks, weeks*.

*^a^Indicated that proteinuria to creatine was reported instead of 24 hours protienuria*.

*^b^Refers to the unit for sCr was μmol/L*.

### Methodological Quality Assessment and Risk of Bias

**Figure 2:**
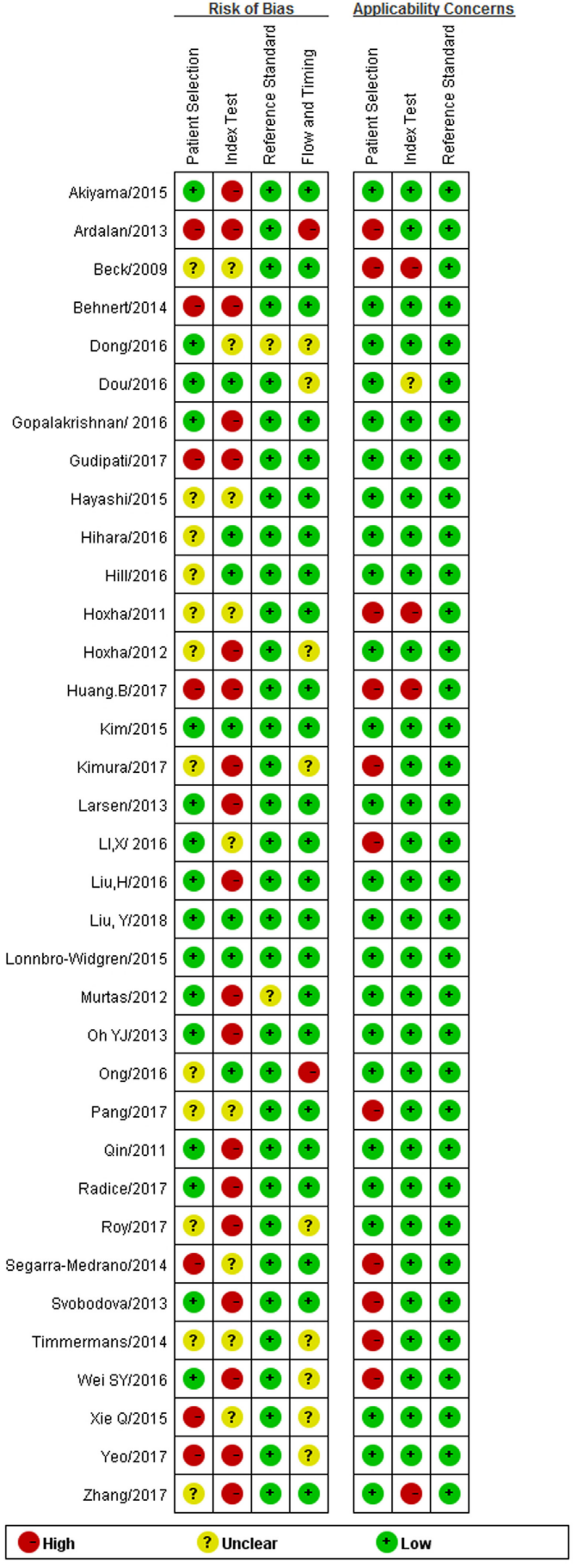
Summary of Quality Assessment of Diagnostic Accuracy Studies-2 criteria.

Generally included studies showed a low concern on applicability of reference standard; studies that were obscure in the setting or interpretation of reference standard were excluded in our analysis. 31.4% of studies did not exclude patients who received IST before serum test or whose proteinuria was below the nephrotic range at baseline, which led to relatively high concerns on applicability of patients’ selection because the interference from IST would influence the immunological activity of patients. Three out of 35 studies did not report a pre-set cutoff value, two of 35 studies tested sPLA2R titer by a new type of method time-resolved FIA, compared to other widely accepted test methods such as ELISA, WB, and IFFT. The unauthorized test methods in these two studies might bring certain concern on the applicability of index test. The risk of bias mainly came from a case–control design in most of the studies. Furthermore, only two studies reported that blinding was applied for the interpretation of index test. 60.0% of studies reported a long time interval between biopsy and serum test or did not provide the related information, which introduced relatively high risk of bias to flow and timing. The methodological quality assessment and risk of bias are summarized in Figure 2.

### Quantitative Synthesis

**Figure 3:**
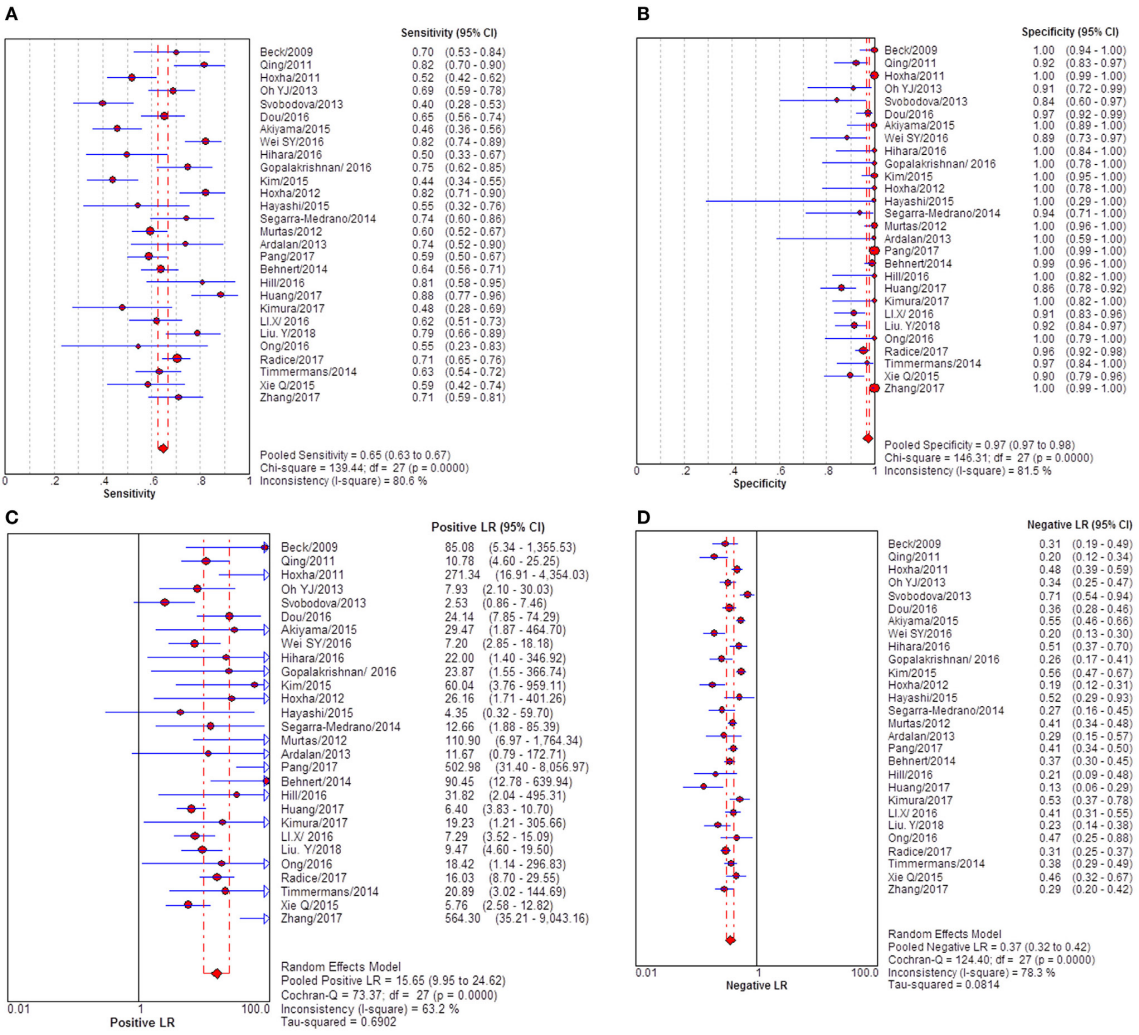
Pooled sensitivity, specificity, PLR, and NLR of serum anti-phospholipase A2 receptor (sPLA2R) for differentiating idiopathic membranous nephropathy (iMN) from non-iMN. **(A)** The pooled sensitivity of sPLA2R for differentiating iMN from non-iMN was 65% (63–67%). **(B)** The pooled specificity of sPLA2R for differentiating iMN from non-iMN was 97% (97–98%). **(C)** The pooled PLR of sPLA2R for differentiating iMN from non-iMN was 15.65 (9.95–24.62). **(D)** The pooled NLR of sPLA2R for differentiating iMN from non-iMN was 0.37 (0.32–0.42). Abbreviations: PLR, positive likelihood ratio, NLR, negative likelihood ratio.

**Figure 4:**
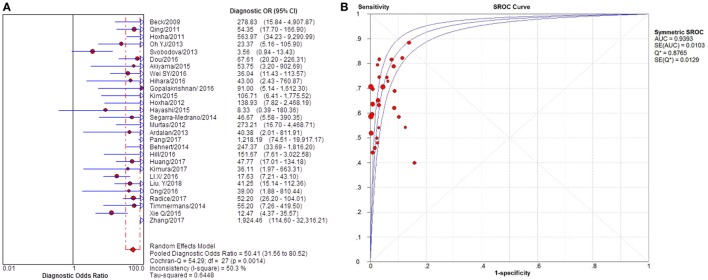
Pooled diagnostic OR and SROC curve of anti-phospholipase A2 receptor (sPLA2R) for differentiating idiopathic membranous nephropathy (iMN) from non-iMN. **(A)** The pooled diagnostic OR of sPLA2R for differentiating iMN from non-iMN was 50.41 (31.56–80.52). **(B)** SROC curve showed that AUC was 0.9393. SROC, summary receiver operating characteristic.

Twenty-eight studies reported the diagnostic value of serum anti-PLA2R for differentiating iMN from non-iMN (Table 2). They indicated a pooled sensitivity of 65% (63–67%), specificity of 97% (97–98%), positive likelihood ratio of 15.65 (9.95–24.62), and negative likelihood ratio of 0.37 (0.32–0.42) with sDOR of 50.41 (31.56–80.52) and AUC of 0.9393. The heterogeneity analysis indicated that Cochran-*Q* = 54.29, df = 27 (*p* = 0.0014), and *I*^2^ for diagnostic OR was 50.3% (Figures 3 and 4). The Spearman correlation coefficient = 0.299 (*p*-value = 0.122), suggesting that there was no threshold effect. SROC plot did not show a curve in the top left corner of the plot, further indicating the lack of threshold effect. Funnel plot showed the existence of asymmetry, which suggested publication bias existing in the studies of serological tests (Figure S1 in Supplementary Material). Meta-regression analyzed covariates including sample size, method types, time interval between biopsy and serum test, whether receiving IST at baseline, and whether excluding patients who were not in nephrotic-range proteinuria for the potential source of heterogeneity, and the result showed that sample size might contribute to the heterogeneity. A subgroup was made with all the studies in which sample size for patients with non-iMN was more than 50, and it showed a diagnostic value of 67.25 (38.56–117.28), *I*^2^ = 47% (Figure S2 in Supplementary Material). Therefore, the heterogeneity was lessened but did not disappear when the restriction for the sample size of included patients with non-iMN was applied.

**Table 2:** Sensitivity and specificity for differentiating iMN with non-iMN by serum anti-PLA2R autoantibodies.

Reference	Method	Cutoff value	iMN:non-iMN	TP	FP	FN	TN	Sensitivity	Specificity
Beck et al. (2)	WB	1:100	37:60	26	0	11	60	0.703 (0.530–0.841)	1.000 (0.940–1.000)
Qing et al. (5)	WB	1:100	60:66	49	5	11	61	0.817 (0.696–0.905)	0.924 (0.832–0.975)
Hoxha et al. (6)	IIFT	1:10	100:260	52	0	48	260	0.520 (0.418–0.621)	1.000 (0.986–1.000)
Oh et al. (7)	WB	1:100	100:23	69	2	31	21	0.690 (0.590–0.779)	0.913 (0.720–0.989)
Svobodova et al. (8)	IIFT	NR	65:19	26	3	39	16	0.400 (0.280–0.529)	0.842 (0.604–0.966)
Dou et al. (9)	ELISA	14 RU/ml	118:111	77	3	41	108	0.653 (0.559–0.738)	0.973 (0.923–0.994)
Akiyama et al. (10)	WB	1:10	100:31	46	0	54	31	0.460 (0.360–0.563)	1.000 (0.888–1.000)
Wei et al. (11)	ELISA	20 U/ml	113:35	93	4	20	31	0.823 (0.740–0.888)	0.886 (0.733–0.968)
Hihara et al. (12)	ELISA	20 U/ml	38:21	19	0	19	21	0.500 (0.334–0.666)	1.000 (0.839–1.000)
Gopalakrishnan et al. (13)	IIFT	1:10	60:15	45	0	15	15	0.750 (0.621–0.853)	1.000 (0.782–1.000)
Kim et al. (14)	ELISA	14 U/ml	93:67	41	0	52	67	0.441 (0.338–0.548)	1.000 (0.946–1.000)
Hoxha et al. (15)	IIFT	NR	73:15	60	0	13	15	0.822 (0.715–0.902)	1.000 (0.782–1.000)
Hayashi et al. (16)	WB	1:100	22:3	12	0	10	3	0.545 (0.322–0.758)	1.000 (0.292–1.000)
Segarra-Medrano et al. (17)	ELISA	15 U/ml	47:17	35	1	12	16	0.745 (0.597–0.861)	0.941 (0.713–0.999)
Murtas et al. (18)	WB	1:100	186:92	111	0	75	92	0.597 (0.523–0.668)	1.000 (0.961–1.000)
Ardalan et al. (19)	IIFT	1:10	23:7	17	0	6	7	0.739 (0.516–0.898)	1.000 (0.590–1.000)
Pang et al. (20)	ELISA	20 U/ml	136:427	80	0	56	427	0.588 (0.501–0.672)	1.000 (0.991–1.000)
Behnert et al. (21)	IIFT	1:10	157:142	100	1	57	141	0.637 (0.557–0.712)	0.993 (0.961–1.000)
Hill et al. (22)	ELISA	2 RU/ml	21:19	17	0	4	19	0.810 (0.581–0.946)	1.000 (0.824–1.000)
Huang et al. (23)	FIA	0.89 mg/ml	52:94	46	13	6	81	0.885 (0.766–0.956)	0.862 (0.775–0.924)
Kimura et al. (24)	IIFT	1:10	25:19	12	0	13	19	0.480 (0.278–0.687)	1.000 (0.824–1.000)
Li et al. (25)	ELISA	20 U/ml	82:82	51	7	31	75	0.622 (0.508–0.727)	0.915 (0.832–0.965)
Liu et al. (26)	ELISA	2.6 RU/ml	57:84	45	7	12	77	0.789 (0.661–0.886)	0.917 (0.836–0.966)
Ong et al. (27)	ELISA	20 RU/ml	11:16	6	0	5	16	0.545 (0.234–0.833)	1.000 (0.991–1.000)
Radice et al. (28)	IIFT	NR	252:227	178	10	74	217	0.706 (0.646–0.762)	0.956 (0.920–0.979)
Timmermans et al. (29)	ELISA	20 RU/ml	109:33	69	1	40	32	0.633 (0.535–0.723)	0.970 (0.842–0.999)
Xie et al. (30)	IIFT	1:10	41:59	24	6	17	53	0.585 (0.421–0.737)	0.898 (0.792–0.962)
Zhang et al. (31)	FIA	2.025 mg/ml	69:389	49	0	20	398	0.710 (0.588–0.813)	1.000 (0.991–1.000)

*TP, true positive; FP, false positive; FN, false negative; TN, true negative; WB, Western blot; IIFT, indirect immunofluorescence test; FIA, fluoroimmunoassay; iMN, idiopathic membranous nephropathy; anti-PLA2R, anti-phospholipase A2 receptor*.

**Figure 5:**
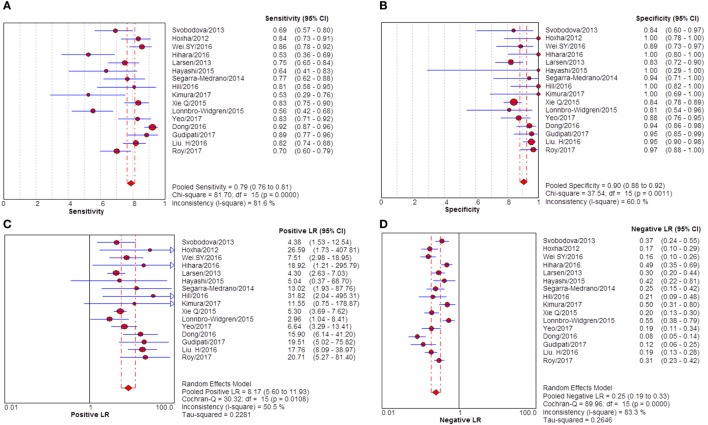
Pooled sensitivity, specificity, positive likelihood ratio (PLR), negative likelihood ratio (NLR) of glomerular PLA2R (gPLA2R) antigen for differentiating idiopathic membranous nephropathy (iMN) from non-iMN. **(A)** The pooled sensitivity of gPLA2R for differentiating iMN from non-iMN was 79% (76–81%). **(B)** The pooled specificity of gPLA2R for differentiating iMN from non-iMN was 90% (88–92%). **(C)** The pooled PLR of gPLA2R for differentiating iMN from non-iMN was 8.17 (5.60–11.93). **(D)** The pooled NLR of gPLA2R for differentiating iMN from non-iMN was 0.25 (0.19–0.33).

**Figure 6:**
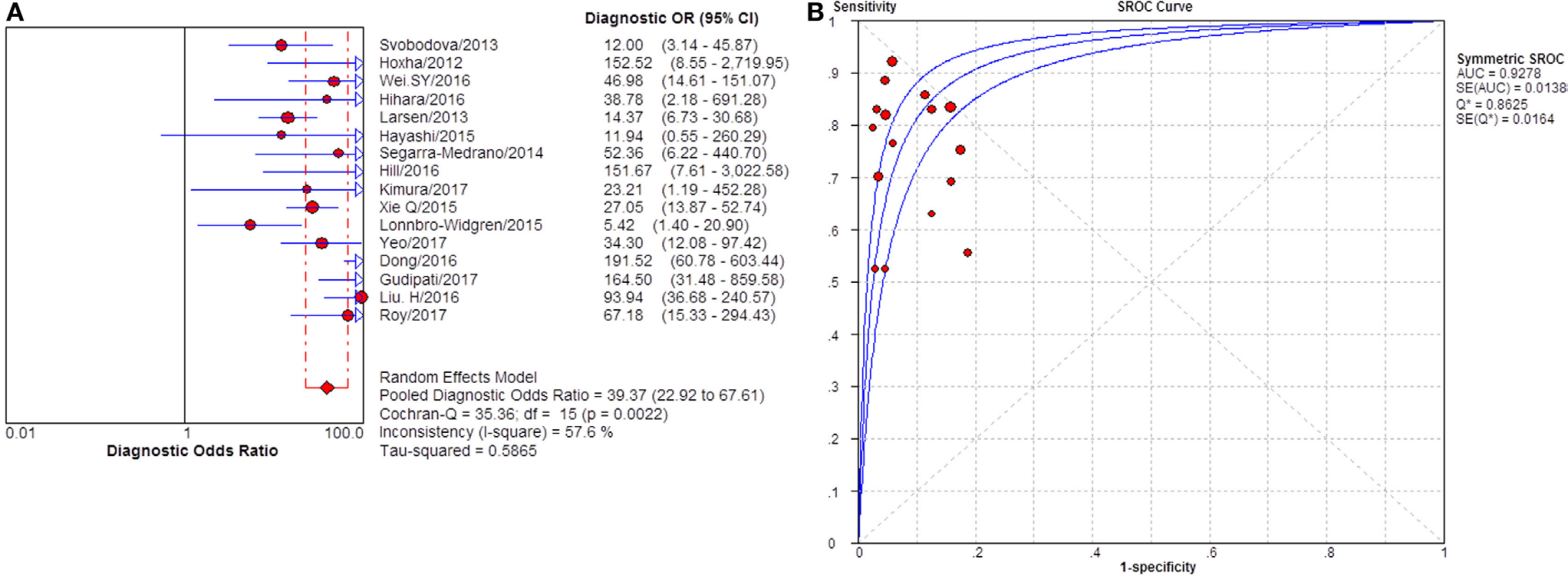
Pooled diagnostic OR and summary receiver operating characteristic (SROC) curve of glomerular PLA2R (gPLA2R) for differentiating idiopathic membranous nephropathy (iMN) from non-iMN. **(A)** The pooled diagnostic OR of gPLA2R for differentiating iMN from non-iMN was 39.37 (22.92–67.61). **(B)** SROC showed that AUC was 0.9278.

Sixteen studies reported the diagnostic value of glomerular PLA2R antigen for differentiating iMN from non-iMN (Table 3). The pooled sensitivity, specificity, positive likelihood ratio, negative likelihood ratio, sDOR, and AUC were 79% (76–81%), 90% (88–92%), 8.17 (5.60–11.93), 0.25 (0.19–0.33), 39.37 (22.18–60.13), and 0.9278 (Figures 5 and 6). Heterogeneity analysis showed that Cochran-*Q* = 35.36; df = 15 (*p* = 0.002), and *I*^2^ for sDOR was 57.6%. The Spearman correlation coefficient = −0.060 (*p*-value = 0.824), suggesting that there is no threshold effect. SROC plot did not show a curve in the top left corner of the plot, further indicating the lack of threshold effect. Funnel plot showed the existence of asymmetry, which suggested publication bias existing in the studies of gPLA2R tests (Figure S3 in Supplementary Material).

**Table 3:** Sensitivity and specificity for differentiating iMN with non-iMN by glomerular deposit of PLA2R.

Reference	Method	Dilution	iMN:non-iMN	TP	FP	FN	TN	Sensitivity	Specificity
Svobodova et al. (8)	IIFT	NR	65:19	45	3	20	16	0.692 (0.566–0.801)	0.842 (0.604–0.966)
Hoxha et al. (15)	IIFT	NR	73:15	61	0	12	15	0.836 (0.730–0.912)	1.000 (0.782–1.000)
Wei et al. (11)	IIFT	1:200	113:35	97	4	16	31	0.858 (0.780–0.917)	0.866 (0.733–0.968)
Hihara et al. (12)	IIFT	1:100	38:17	20	0	18	17	0.526 (0.358–0.690)	1.000 (0.805–1.000)
Larsen et al. (33)	IIFT	1:50	85:80	64	14	21	66	0.753 (0.647–0.840)	0.825 (0.724–0.901)
Hayashi et al. (16)	IHC	1:400	22:3	4	0	8	3	0.636 (0.407–0.828)	1.000 (0.292–1.000)
Segarra-Medrano et al. (17)	IHC	NR	47:17	36	1	11	16	0.766 (0.620–0.877)	0.941 (0.713–0.999)
Hill et al. (22)	IIFT	1:500	19:19	17	0	4	19	0.810 (0.581–0.946)	1.000 (0.824–1.000)
Kimura et al. (24)	IFFT	NR	19:10	10	0	9	10	0.526 (0.289–0.756)	1.000 (0.692–1.000)
Xie et al. (30)	IIFT	1:500	102:165	86	26	17	139	0.835 (0.749–0.901)	0.842 (0.778–0.894)
Lonnbro-Widgren et al. (32)	IIFT	1:8000	63:16	35	3	28	13	0.556 (0.425–0.681)	0.813 (0.544–0.960)
Yeo et al. (34)	IHC	1:2000	59:56	49	7	10	49	0.831 (0.710–0.916)	0.875 (0.759–0.948)
Dong et al. (35)	IHC	1:800	179:69	165	4	14	65	0.922 (0.872–0.957)	0.942 (0.858–0.984)
Gudipati et al. (36)	IHC	NR	51:44	47	2	6	42	0.887 (0.770–0.957)	0.955 (0.845–0.994)
Liu et al. (37)	IIFT	1:100	122:130	100	6	22	124	0.820 (0.740–0.883)	0.954 (0.902–0.983)
Roy et al. (38)	IHC	NR	94:59	66	2	28	57	0.702 (0.599–0.792)	0.966 (0.883–0.996)

*TP, true positive; FP, false positive; FN, false negative; TN, true negative; WB, Western blot; IIFT, indirect immunofluorescence test*.

### Subgroup Analysis

**Figure 7:**
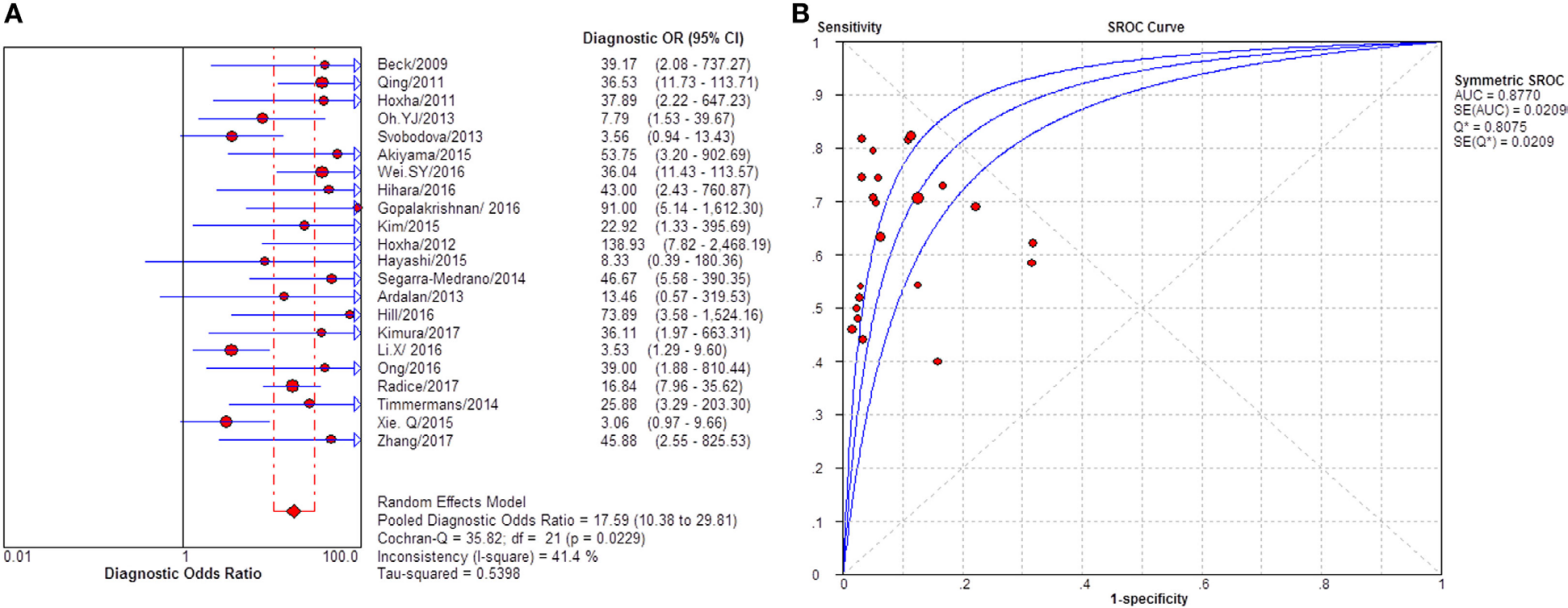
Pooled diagnostic OR and summary receiver operating characteristic (SROC) curve of serum anti-phospholipase A2 receptor (sPLA2R) for differentiating idiopathic membranous nephropathy (iMN) from SMN. **(A)** The pooled diagnostic OR of sPLA2R for differentiating iMN from secondary membranous nephropathy (SMN) was 17.59 (10.38–29.81). **(B)** SROC showed that AUC was 0.8770.

Twenty-two studies reported the diagnostic value of sPLA2R for differentiating iMN from SMN. They indicated a pooled sensitivity of 65% (62–67%), specificity of 91% (88–94%), positive likelihood ratio of 5.91 (3.81–9.16), and negative likelihood ratio of 0.39 (0.33–0.46) with sDOR of 17.59 (10.38–29.81) and AUC of 0.8770 (Figure 7). *I*^2^ for diagnostic OR was 41.4%, Cochran-*Q* = 35.82, df = 21 (*p*-value = 0.023).

**Figure 8:**
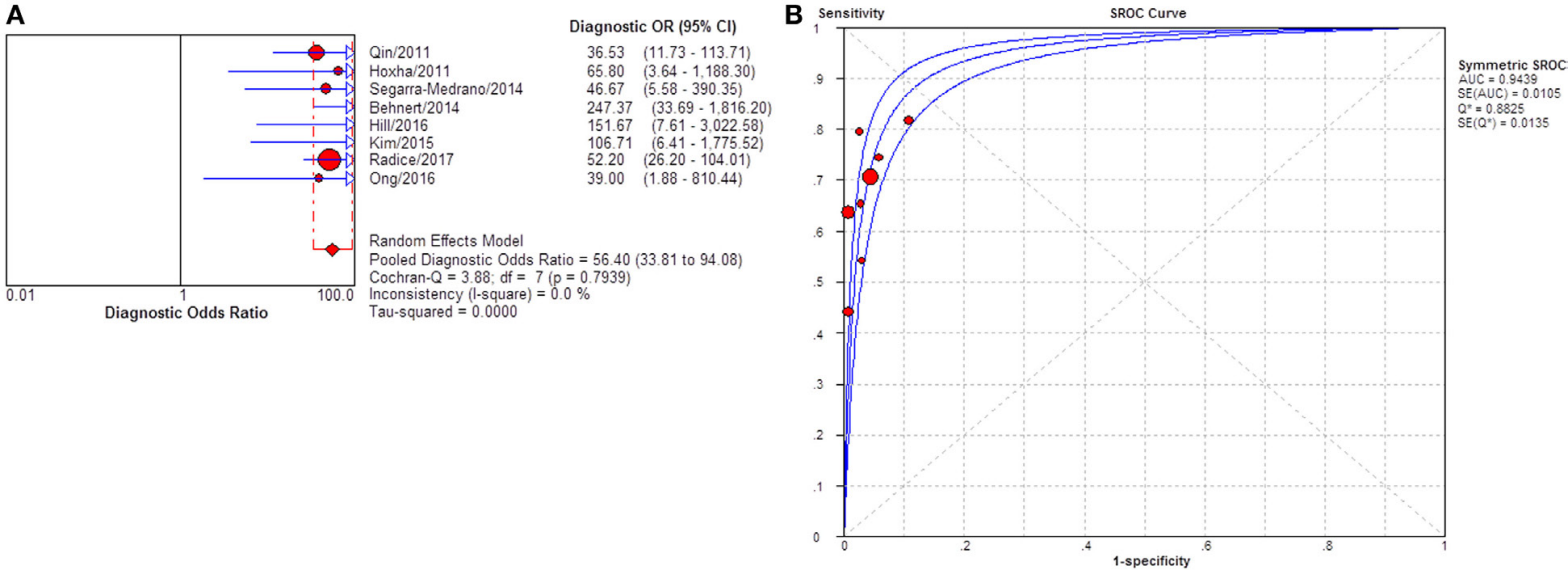
Pooled diagnostic OR and summary receiver operating characteristic (SROC) curve of serum anti-phospholipase A2 receptor (sPLA2R) for studies that only enrolled patients with NRP at baseline. **(A)** The pooled diagnostic OR of sPLA2R for studies that only enrolled patients with NRP at baseline was 56.40 (33.81–94.08). No heterogeneity was detected. **(B)** SROC showed that AUC was 0.9439. NRP, nephrotic range proteinuria.

**Figure 9:**
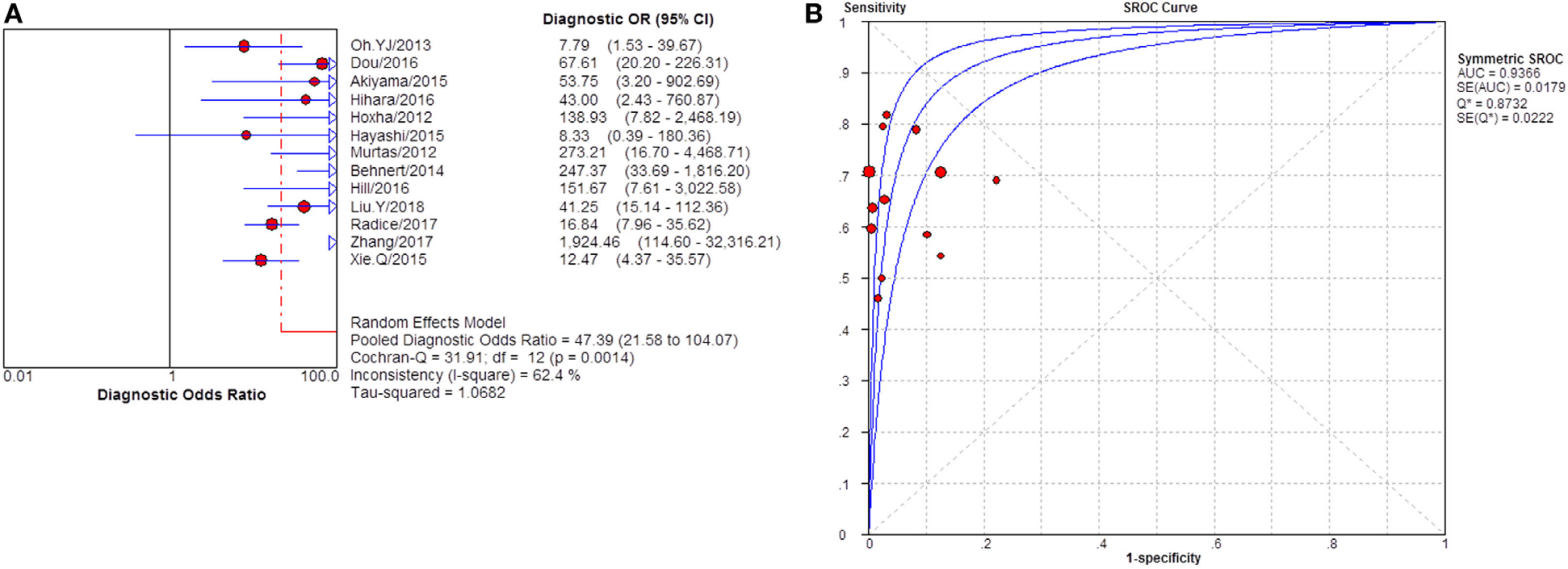
Pooled diagnostic OR and summary receiver operating characteristic (SROC) curve of serum anti-phospholipase A2 receptor (sPLA2R) for studies that only enrolled patients with no IST at baseline. **(A)** The pooled diagnostic OR of sPLA2R for studies that only enrolled patients with no IST at baseline was 47.39 (21.58–104.07). The heterogeneity was 62.4%. **(B)** SROC showed that AUC was 0.9366. IST, immunosuppressive therapy.

**Figure 10:**
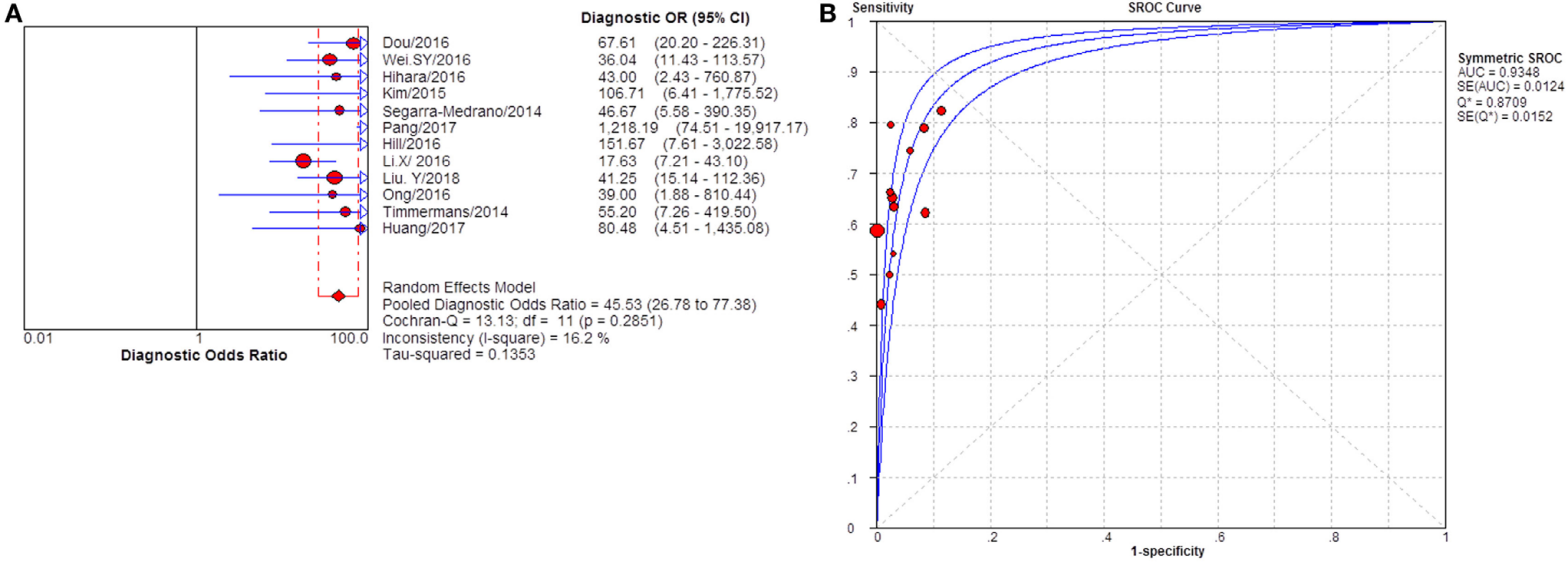
Pooled diagnostic OR and summary receiver operating characteristic (SROC) curve of serum anti-phospholipase A2 receptor (sPLA2R) for studies that used ELISA as test method. **(A)** The pooled diagnostic OR of sPLA2R for studies that used ELISA as test method was 45.53 (26.78–77.38). The heterogeneity was insignificant. **(B)** SROC showed that AUC was 0.9348. ELISA, enzyme-linked immunosorbent assay.

**Figure 11:**
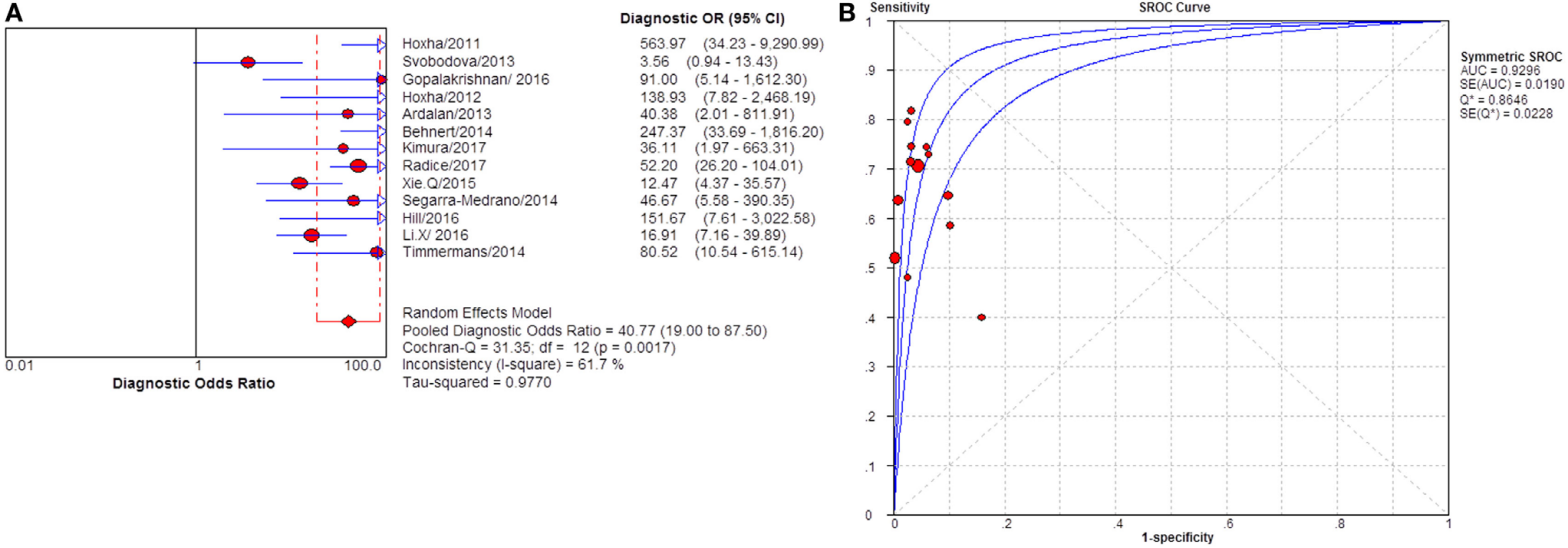
Pooled diagnostic OR and summary receiver operating characteristic (SROC) curve of serum anti-phospholipase A2 receptor (sPLA2R) for studies that used IIFT as test method. **(A)** The pooled diagnostic OR of sPLA2R for studies that used IIFT as test method was 40.77 (19.00–87.50). The heterogeneity was 61.7% **(B)** SROC showed that AUC was 0.9296. IIFT, indirect immunofluorescence test.

**Figure 12:**
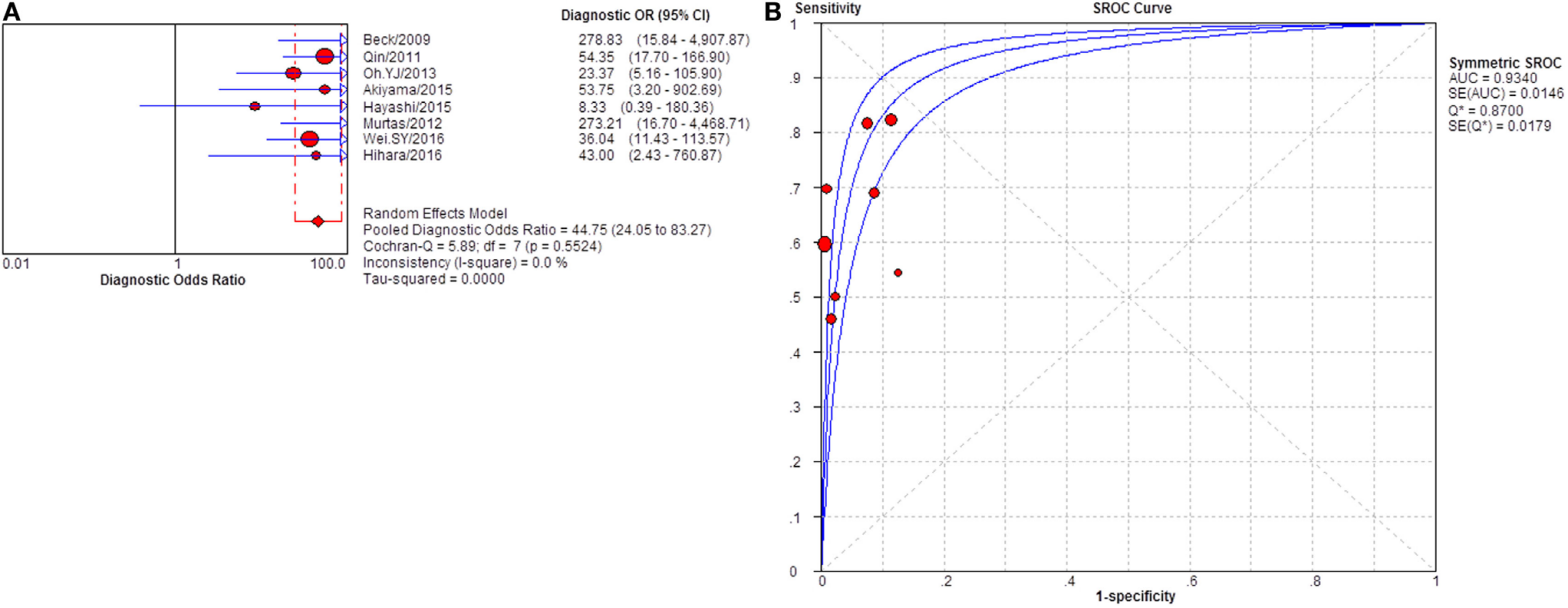
Pooled diagnostic OR and summary receiver operating characteristic (SROC) curve of serum anti-phospholipase A2 receptor (sPLA2R) for studies that used western blot as test method. **(A)** The pooled diagnostic OR of sPLA2R for studies that used western blot as test method was 44.75 (24.05–83.27). The heterogeneity was 61.7% **(B)** SROC showed that AUC was 0.9340.

Severity of proteinuria and interference of IST are factors that would affect the immunological activity and disease status. In the assessment of applicability, both severity of proteinuria and interference from IST were the factors that might bring risk of bias on the applicability of patient selection, and therefore the effect from each factor was explored by subgroup analysis separately. First, a subgroup included studies that set a specific criteria on patient selection and in which only patients with nephrotic-range proteinuria at the time of sampling were enrolled (Table S1 in Supplementary Material). A pooled diagnostic OR for studies only including patients with nephrotic-range proteinuria was 56.40 (33.81–94.08). The heterogeneity analysis showed that *I*^2^ = 0.0%, Cochran-*Q* = 3.88, df = 7 (*p* = 0.794) (Figure 8). The heterogeneity disappeared after considering the covariate of ratio of patients with nephrotic-range proteinuria at baseline, which suggested that it might be the source of heterogeneity. Moreover, the interference of IST might cover up the real level of sPLA2R and gPLA2R deposit resulting from the disease-related pathological process. Studies that specified that all the serums were collected from patients before receiving any IST were analyzed as a subgroup (Table S2 in Supplementary Material) and the result for heterogeneity analysis indicated that *I*^2^ = 62.4%, Cochran-*Q* = 31.9, df = 12 (*p* = 0.0014) (Figure 9). The consistency of heterogeneity suggested that the ratio of patients who received IST before serum collection did not contribute to the heterogeneity in this analysis. Finally, a subgroup analysis for the test method showed that the use of different assay methods for testing serum level of anti-PLA2R was one of the causes of heterogeneity (Figures 10–12). After pooling all the results of studies that using ELISA as the test method, the heterogeneity turned to be *I*^2^ = 16.2%, *p* = 0.2851 (Table S3 in Supplementary Material). For all the studies that used WB as the single test method, the heterogeneity analysis showed that *I*^2^ = 0.0%, *p* = 0.5524 (Table S4 in Supplementary Material). The heterogeneity of the subgroup analysis for ELISA and WB disappeared, which suggested that the test method might be the source of heterogeneity.

## Discussion

Few meta-analyses evaluating diagnostic test accuracy of anti-PLA2R have been reported previously: Dai et al. performed a diagnostic accuracy meta-analysis for sPLA2R and gPLA2R in 2015; however, they included eight meeting abstracts for statistical synthesis, which compromised the fidelity of the analysis because of the low standard for study inclusion (39). Besides, two studies that did not focus on the diagnostic performance of anti-PLA2R and also lacked sufficient data for quantitative synthesis were also included. Yeo et al. also reported the result of diagnostic test for anti-PLA2R; however, only eight studies were included in total and literature searches were only updated until the year of 2016 (34). In this study, we did a comprehensive literature search in common databases without language restriction, and the database search was updated until February 2018. As a result, 35 records were identified under a strict inclusion criterion. Twenty-three more records were included compared with the latest available meta-analysis for the diagnostic performance of anti-PLA2R. Also, we excluded conference abstracts and repeated publication on the same cohort to avoid unnecessary bias and to improve the overall quality of the studies included. The source of heterogeneity was fully explored by meta-regression and subgroup analysis. Our analysis showed that both anti-PLA2R autoantibodies and glomerular deposit of PLA2R antigen demonstrated a good diagnostic accuracy in differentiating iMN from non-iMN or SMN. Heterogeneity mainly came from the test method and ratio of patients with NRP at baseline. The prevalence of serum PLA2R reported by studies in different area varies from 57 to 82% (5, 6, 10, 13, 25, 40, 41). In this study, pooled sensitivity of serum anti-PLA2R and gPLA2R for differentiating iMN from non-iMN are 65 and 79%, respectively. The relatively low sensitivity might bring limitations on the interpretation of test result especially with a negative result. Apparently, serological test for anti-PLA2R is not a suitable option for screening test, and a negative test result leads to the need for renal biopsy and further search for potential secondary causes. However, both test for serum PLA2R and PLA2R antigen has high specificity, which means that a positive result indicates a high likely diagnosis of iMN. Its high positive predictive value helps the exclusion of non-MN diseases with a positive test result.

The limitations of this study are discussed subsequently. First of all, the heterogeneity of included studies was significant although attempts were made to find the source of heterogeneity by subgroup analysis and meta-regression. Besides, asymmetry was observed in the funnel plot with more studies appeared toward right (indicating higher odds ratio) in the bottom of the graph, which indicated the possibility that some studies might be missing on the left. The exclusion of conference abstracts and gray literature in study selection might account for the asymmetry; however this exclusion criterion was set to improve the overall quality of the included studies. Furthermore, concern on the applicability of patient selection might be relatively high because of the prevalent case–control design in included studies. A proportion of studies also did not set a strict enrollment criterion for patient selection which failed to minimize the interference from the severity of proteinuria and the use of IST before index test. But subgroup analysis was conducted according to the severity of proteinuria and whether or not receiving IST to specify the feature of target patients.

Renal biopsy is considered as the gold standard for diagnosing MN for a long time. However, our current standard to discriminate iMN from SMN might be confused by some subjective factors. Searching for secondary causes could be omitted or there may be a delayed appearance or detection of clinical presentation or evidence of secondary etiology. Under these circumstances, looking for a better marker to discriminate iMN from SMN that could be interpreted objectively is of great clinical significance (42). The discovery and developing insights toward the role of anti-PLA2R in MN is a milestone in our understanding toward the pathological mechanism of iMN (4). It is noteworthy that sPLA2R and gPLA2R demonstrated a high specificity in iMN, in contrast with in SMN or other non-MN GN, which indicated that anti-PLA2R antibodies were a highly possible cause of glomerular pathology rather than the consequence of proteinuria or glomerular injury. The potential etiological role of anti-PLA2R in iMN and the development of serological assay for anti-PLA2R bring the possibility of non-invasive diagnosis for iMN (43). Nevertheless, question remained about a non-negligible percentage of patients with iMN showing negative test result for serum anti-PLA2R. The negative cases in iMN could be explained by possible existence of other pathogenesis-related auto-antigens such as thrombospondin type1 domain-containing 7A and neutral endopeptidase (44). Besides, the recognition of antigen is strictly configuration-dependent and only reacts with reduction-sensitive epitope (2). Therefore, it is possible that there are other undiscovered cryptic epitopes. Due to a relatively low sensitivity, routine serum test for the anti-PLA2R level is recommended for patient with proteinuria or nephrotic syndrome of unknown etiology in our clinical practice, but a negative result is not able to exclude iMN, which warrants further evaluation by renal biopsy. The sensitivity of gPLA2R was 79%, which was a litter higher than sPLA2R. The combined measurement and interpretation of sPLA2R and gPLA2R might boost the overall performance of the diagnostic value, which worth further exploration. On the other aspect, serological test for anti-PLA2R is known to be highly specific, even though there were still reports about positive results in the test for anti-PLA2R in SMN (1), which is consistent with a pooled specificity of 91% reported in this study. However, there were also studies reporting that the positive test for serum anti-PLA2R were proved to be iMN with superimposed but unrelated hepatitis virus infection or cancer (5, 45), and some scholars advocated the value of anti-PLA2R in excluding non-MN diseases, because no positive result in the test for serum anti-PLA2R has been found in patients with proteinuric condition other than MN (46). More evidence and understanding toward the exact pathogenic role of PLA2R in MN need to be accumulated to provide a thorough rationale for the applicability of PLA2R related test (5).

Based on the result of this meta-analysis and existing evidence, we recommended that the interpretation should be combined with renal biopsy and clinical findings before the next milestone of our comprehension toward PLA2R is achieved. Studies with stricter enrollment criteria to reduce the interference from IST and to ensure the enrolled patients are in an immunologically active stage of the disease would be helpful in giving us a more accurate view on the prevalence of anti-PLA2R in iMN, although the post-treatment measurement of sPLA2R might have its potential value in monitoring disease activity and serve as a guide for therapeutic strategy (47, 48). Future research should focus on evaluating the diagnostic value of anti-PLA2R IgG 4 subtype, which is the prominent subtype of immunoglobulin in iMN and the significance of the combining serum anti-PLA2R and gPLA2R deposit in diagnosing iMN (40).

## Conclusion

Both sPLA2R and gPLA2R demonstrated a good diagnostic accuracy in differentiating iMN and non-iMN. The positive test for sPLA2R is highly indicative for the diagnosis of iMN. We recommend that the conduction and interpretation of test for anti-PLA2R and gPLA2R should be combined with renal biopsy and calibrated according to specific clinical scenario.

## Author Contributions

WL took the major in conceptualization, literature search and review, article drafting, and writing. YZ contributed by editing and reviewing. PF contributed by general supervision, reviewing, and validation.

## Conflict of Interest Statement

The authors declare that the research was conducted in the absence of any commercial or financial relationships that could be construed as a potential conflict of interest.
