# Supplementary material for: Diagnostic Test Accuracy of Serum Anti-PLA2R Autoantibodies and Glomerular PLA2R Antigen for Diagnosing Idiopathic Membranous Nephropathy: An Updated Meta-Analysis
Source: Front Med (Lausanne). 2018 Apr 26;5:101. doi: 10.3389/fmed.2018.00101 (PMC5932148; doi:10.3389/fmed.2018.00101)

Figure S1. Funnel plot for the analysis of sPLA2R in differentiating iMN from non-iMN.

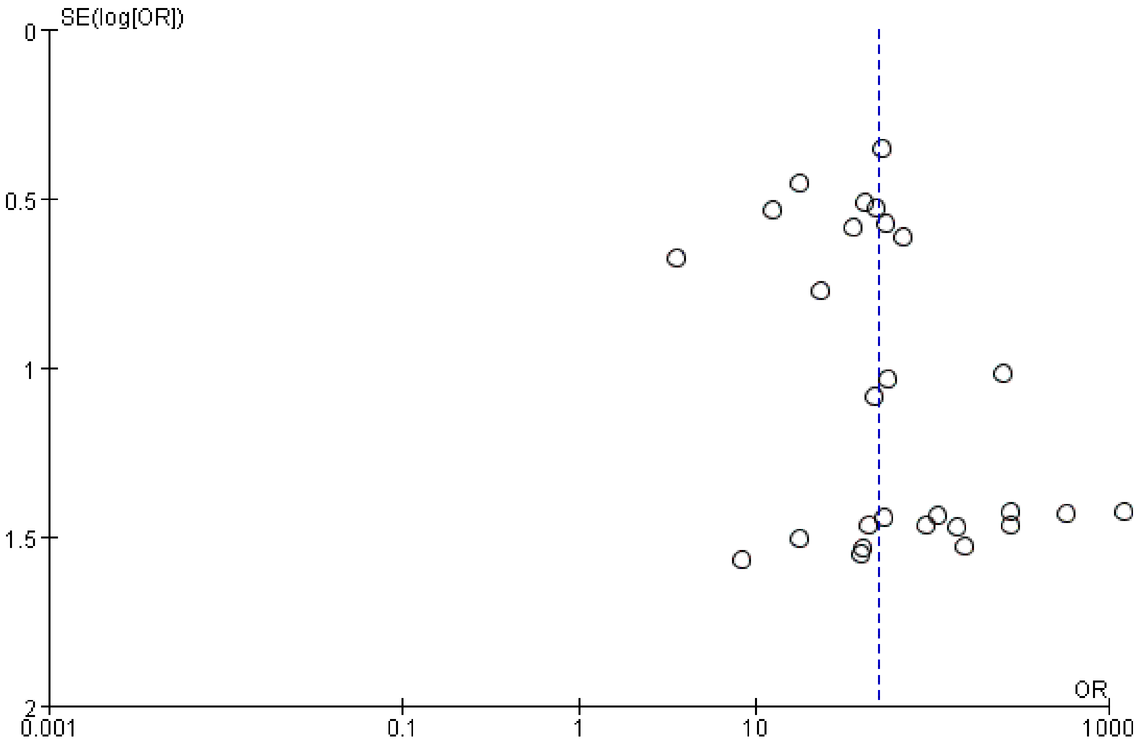

Figure S2. Pooled Diagnostic OR for studies with sample size of patients with non-iMN above fifty.

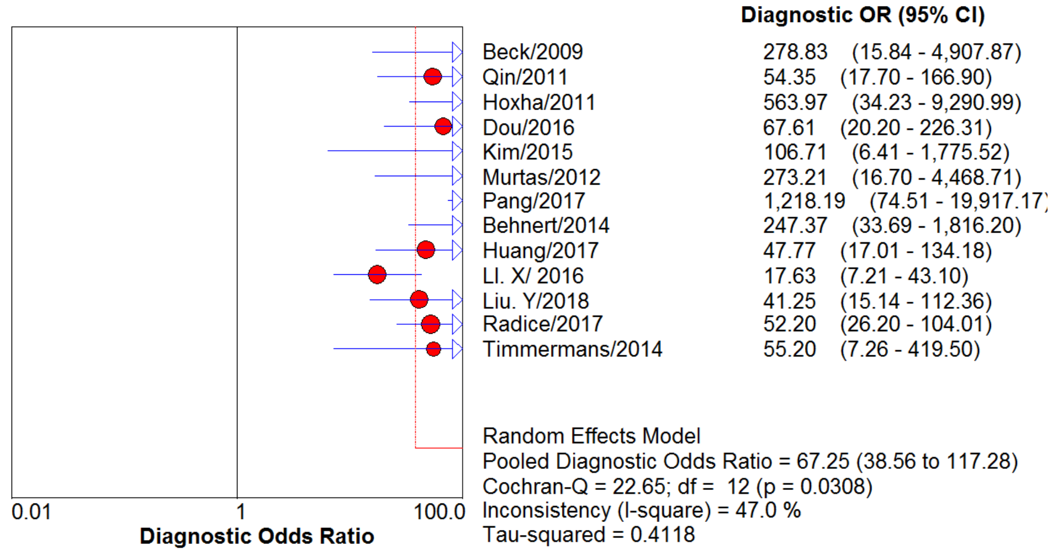

Figure S3. Funnel plot for the analysis of gPLA2R in differentiating iMN from non-iMN.

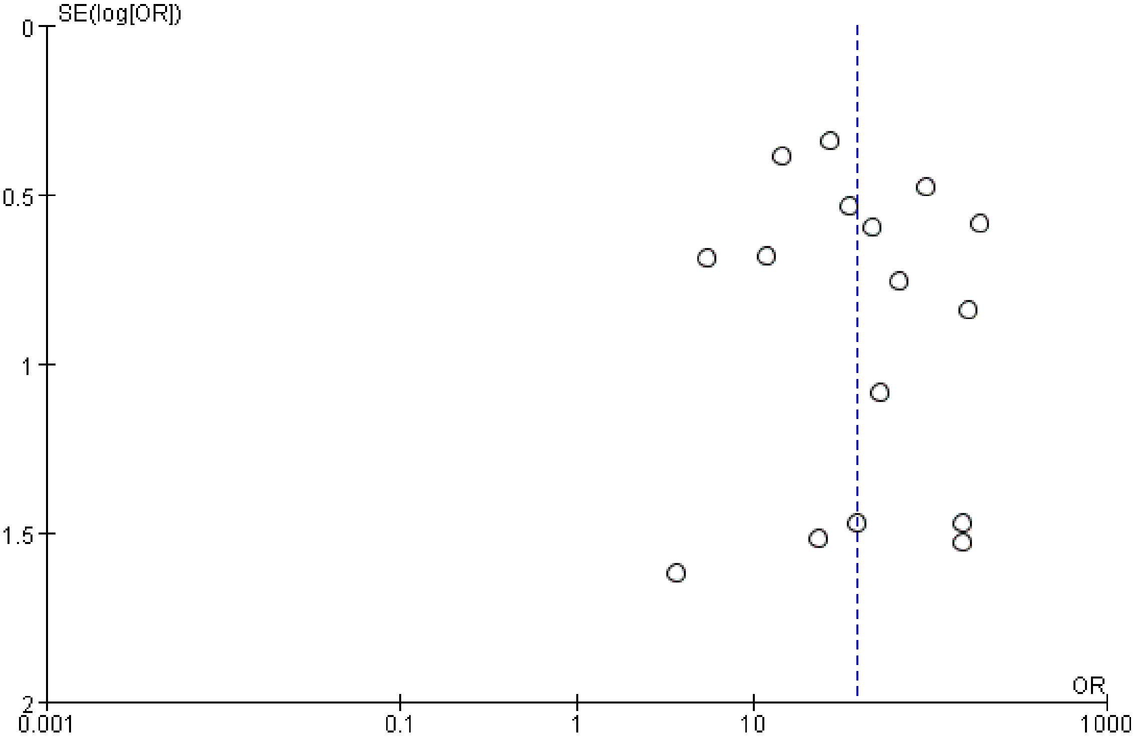

Supplement: Supplementary file 1 [file Image_1.PDF]
