# Supplementary material for: Diagnostic Test Accuracy of Serum Anti-PLA2R Autoantibodies and Glomerular PLA2R Antigen for Diagnosing Idiopathic Membranous Nephropathy: An Updated Meta-Analysis
Source: Front Med (Lausanne). 2018 Apr 26;5:101. doi: 10.3389/fmed.2018.00101 (PMC5932148; doi:10.3389/fmed.2018.00101)
Supplement: Supplementary file 2 [file Table_1.PDF]

Table S1. TP, FP, TN and FN for studies with patients in NRP.

author/year	test method	cutoff value	IMN:SMN	TP	FP	FN	TN
Qin/2011	WB	1:100	60:46	49	5	11	41
Hoxha/2011	IFA	1:10	35:17	23	0	12	17
Segarra-Medrano/2014	ELISA	15U/ml	47:17	35	1	12	16
Behnert/2014	IIFT	1:10	157:142	100	1	57	141
Hill/2016	ELISA	2RU/ml	21:19	17	0	4	19
Kim/2015	ELISA	14U/ml	93:67	41	0	52	67
Radice/2017	IFFT	NR	252:227	178	10	74	217
Ong/2016	ELISA	20 RU/mL	11:16	6	0	5	16

Table S2. TP, FP, TN and FN for studies only enrolling patients with no IST at baseline.

author/year	test method	cutoff value	IMN:non-IMN	TP	FP	FN	TN
Hoxha/2011	IFA	1:10	36:17	21	0	15	17
Oh,YJ/2013	WB	1:100	100:9 （SMN）	69	2	31	7
Dou/2016	ELISA	14RU/ml	118:111 (non-SMN)	77	3	41	108
Akiyama/2015	WB	1:10	100:31 (SMN)	46	0	54	31
Hihara/2016.	ELISA	20U/ml	38:21	19	0	19	21
Hoxha/2012	IIFT	NR	73:15	60	0	13	15
Hayashi/2015	WB	1:100	22:3	12	0	10	3
Murtas/2012	WB	1:100	186:92	111	0	75	92
Behnert/2014	IIFT	1:10	157:142	100	1	57	141
Hill/2016	ELISA	2RU/ml	21:19	17	0	4	19
Liu, Y/2018	ELISA	2.6 RU/ml	57:84	45	7	12	77
Radice/2017	IFFT	NR	252:72	178	9	74	63
Zhang/2017	FIA	2.025mg/ml	69:389	49	0	20	398
Xie Q/2015	IFFT	1:10	41:59	24	6	17	53

Table S3. TP, FP, TN and FN for studies using ELISA as test method.

author/year	test method	cutoff value	IMN:non IMN	TP	FP	FN	TN

Dou/2016	ELISA	14RU/ml	118:111	77	3	41	108
Wei SY/2016	ELISA	20U/ml	113:35	93	4	20	31
Hihara/2016	ELISA	20U/ml	38:21	19	0	19	21
Kim/2015	ELISA	14U/ml	93:67	41	0	52	67
Segarra-Medrano/2014	ELISA	15U/ml	47:17	35	1	12	16
Pang/2017	ELISA	20U/ml	136:427	80	0	56	427
Hill/2016	ELISA	2RU/ml	21:19	17	0	4	19
Li,X/ 2016	ELISA	20U/ml	82:82	51	7	31	75
Liu, Y/2018	ELISA	2.6 RU/ml	57:84	45	7	12	77
Ong/2016	ELISA	20 RU/mL	11:16	6	0	5	16
Timmermans/2014	ELISA	20 RU/mL	109:33	69	1	40	32
Huang/2017	ELISA	20U/ml	39:20	26	0	13	20

Table S4. TP, FP, TN and FN for studies using western blot as test method.

author/year	test method	cutoff value	IMN:non IMN	TP	FP	FN	TN
Beck/2009	WB	1:100	37:60	26	0	11	60
Qin/2011	WB	1:100	60:66	49	5	11	61
Oh YJ/2013	WB	1:100	100:23	69	2	31	21
Akiyama/2015	WB	1:10	100:31	46	0	54	31
Hayashi/2015	WB	1:100	22:3	12	0	10	3
Murtas/2012	WB	1:100	186:92	111	0	75	92
Wei SY/2016	WB	1:10	113:35	93	4	20	31
Hihara/2016	WB	1:10	38:21	19	0	19	21
